# Beta-Cryptoxanthin Inhibits Lipopolysaccharide-Induced Osteoclast Differentiation and Bone Resorption via the Suppression of Inhibitor of NF-κB Kinase Activity

**DOI:** 10.3390/nu11020368

**Published:** 2019-02-10

**Authors:** Narumi Hirata, Ryota Ichimaru, Tsukasa Tominari, Chiho Matsumoto, Kenta Watanabe, Keita Taniguchi, Michiko Hirata, Sihui Ma, Katsuhiko Suzuki, Florian M.W. Grundler, Chisato Miyaura, Masaki Inada

**Affiliations:** 1 Cooperative Major of Advanced Health Science, Tokyo University of Agriculture and Technology, 2-24-16 Nakacho, Koganei, Tokyo 184-8588, Japan; narumi-hirata@st.go.tuat.ac.jp (N.H.); s184757q@st.go.tuat.ac.jp (R.I.); miyaura@cc.tuat.ac.jp (C.M.); 2 Department of Biotechnology and Life Science, Tokyo University of Agriculture and Technology, 2-24-16 Nakacho, Koganei, Tokyo 184-8588, Japan; tominari@cc.tuat.ac.jp (T.T.); c-matsu@cc.tuat.ac.jp (C.M.); s186946u@st.go.tuat.ac.jp (K.T.); hirata@cc.tuat.ac.jp (M.H.); 3 Institute of Global Innovation Research, Tokyo University of Agriculture and Technology, 2-24-16 Nakacho, Koganei, Tokyo 184-8588, Japan; kenta-w@cc.tuat.ac.jp (K.W.); grundler@uni-bonn.de (F.M.W.G.); 4 Graduate School of Sport Sciences, Waseda University, 2-579-15 Mikajima Tokorozawa-shi, Tokyo 359-1192, Japan; masihui@toki.waseda.jp; 5 Faculty of Sport Sciences, Waseda University, 2-579-15 Mikajima Tokorozawa-shi, Tokyo 359-1192, Japan; katsu.suzu@waseda.jp; 6 Institute of Crop Science and Resource Conservation, University of Bonn, Karlrobert-Kreiten-Strasse 13, 53115 Bonn, Germany

**Keywords:** beta-cryptoxanthin, bone resorption, lipopolysaccharide, periodontitis, osteoclast differentiation

## Abstract

Beta-cryptoxanthin (β-cry) is a typical carotenoid found abundantly in fruit and vegetables such as the Japanese mandarin orange, persimmon, papaya, paprika, and carrot, and exerts various biological activities (e.g., antioxidant effects). We previously reported that β-cry suppressed lipopolysaccharide (LPS)-induced osteoclast differentiation via the inhibition of prostaglandin (PG) E_2_ production in gingival fibroblasts and restored the alveolar bone loss in a mouse model for periodontitis in vivo. In this study, we investigated the molecular mechanism underlying the inhibitory effects of β-cry on osteoclast differentiation. In mouse calvarial organ cultures, LPS-induced bone resorption was suppressed by β-cry. In osteoblasts, β-cry inhibited PGE_2_ production via the downregulation of the LPS-induced mRNA expression of cyclooxygenase (COX)-2 and membrane-bound PGE synthase (mPGES)-1, which are PGE synthesis-related enzymes, leading to the suppression of receptor activator of NF-κB ligand (RANKL) mRNA transcriptional activation. In an in vitro assay, β-cry directly suppressed the activity of the inhibitor of NF-κB kinase (IKK) β, and adding ATP canceled this IKKβ inhibition. Molecular docking simulation further suggested that β-cry binds to the ATP-binding pocket of IKKβ. In Raw264.7 cells, β-cry suppressed RANKL-mediated osteoclastogenesis. The molecular mechanism underlying the involvement of β-cry in LPS-induced bone resorption may involve the ATP-competing inhibition of IKK activity, resulting in the suppression of NF-κB signaling.

## 1. Introduction

Bone homeostasis is precisely controlled by maintaining a balance between bone formation and bone resorption, and imbalances in bone remodeling result in various skeletal diseases, such as osteoporosis and periodontitis. Osteoclasts are multinucleated giant cells derived from hematopoietic stem cells that exhibit bone-resorbing activity, whereas osteoblasts are derived from mesenchymal stem cells and are responsible for bone formation. Receptor activator of NF-κB ligand (RANKL) is a key molecule involved in the differentiation of osteoclast precursors into multinucleated osteoclasts [1]. RANKL expressed on the cell surface of osteoblasts binds to RANK expressed on osteoclast precursor cells, and this RANKL–RANK interaction induces osteoclast differentiation and bone resorption via NF-κB signaling. The proinflammatory molecules, including lipopolysaccharide (LPS) and interleukin (IL)-1, promote prostaglandin (PG) E_2_ production and subsequently induce RANKL expression in osteoblasts, leading to osteoclast differentiation [2,3,4,5,6]. PGE_2_ is mainly produced by osteoblasts and fibroblasts under inflammatory conditions [7] and is synthesized by cyclooxygenase (COX)-2 and membrane-bound PGE synthase (mPGES)-1 in the arachidonic acid cascade. We previously reported that PGE_2_ bound to its receptor EP4 expressed on osteoblasts, leading to osteoclastic bone resorption associated with inflammation [3,4].

Periodontitis is a local bone disease associated with inflammation caused by the infection of Gram-negative bacteria, and the development of periodontitis results in alveolar bone resorption and tooth loss. LPS, an outer membrane component of Gram-negative bacteria, is a pathogen of periodontitis and is identified as a ligand for toll-like receptor (TLR) 4 [8]. Our previous study reported that LPS–TLR4 signaling stimulated PGE_2_ production through the upregulation of COX-2 and mPGES-1 mRNA expression in osteoblasts, resulting in osteoclastic bone resorption [5,9]. We also reported that the local injection of LPS into lower gingiva induced alveolar bone loss in mice; however, LPS injection failed to induce alveolar bone loss in mPGES1-deficient mice [5]. PGE_2_ is therefore an essential factor for LPS-induced inflammatory bone resorption.

Carotenoids are abundantly contained in fruits and vegetables, and exert beneficial activity. Beta-cryptoxanthin (β-cry) is a typical carotenoid found abundantly in Japanese mandarin orange, persimmon, papaya, paprika, and carrot. β-cry has been reported to possess several beneficial functions, such as antioxidant, cancer-preventive effects, and anti-metabolic syndrome effects [10,11,12]. Park et al. [13] showed that the daily oral administration of β-cry prevented the progression of osteoarthritis and inhibited proinflammatory cytokines in mice. Ozaki et al. [14] reported that the daily intake of β-cry suppressed osteoclast differentiation via the repression of the NF-κB pathway, and ameliorated estrogen deficiency-induced bone loss in ovariectomized mice. Uchiyama et al. [15,16] showed that β-cry promoted osteoblastic bone formation and mineralization via the upregulated expression of insulin growth factor (IGF)-1, transforming growth factor (TGF)-β, collagen type 1 alpha 1 (Col1a1), runt-related transcription factor (RUNX) 2, and alkaline phosphatase in MC3T3-E1 cells. Regarding the molecular mechanisms, Yamaguchi et al. [17,18] reported that β-cry stimulated bone formation via the activation of TGF-β signaling, and β-cry bound to RXR or orphan receptors in the nucleus in order to regulate bone formation-related genes. Both mitogen-activated protein kinase (MAPK) and protein kinase (PK) C may also be target molecules of β-cry in osteoblastic bone formation [15]. Uchiyama and Yamaguchi [19,20,21] further reported that β-cry inhibited osteoclast differentiation and induced apoptosis in mature osteoclasts. We previously showed that β-cry suppressed PGE_2_ production in murine primary gingival fibroblasts and inhibited the LPS-induced bone loss of mandibular alveolar bone in a mouse model for periodontitis [22]; however, the target molecule of β-cry in bone resorption is unclear.

In the present study, we clarified the molecular mechanisms underlying the involvement of β-cry in osteoclast differentiation.

## 2. Materials and Methods

### 2.1. Animals and Reagents

Newborn, 6-week-old male mice of the *ddY* strain were obtained from Japan SLC Inc. (Shizuoka, Japan). All procedures were performed in accordance with the institutional guidelines for animal research. β-cry (purity: ≥97%) was obtained from FUJIFILM Wako Pure Chemical Corporation (Osaka, Japan). LPS from *Escherichia coli* was provided by Sigma-Aldrich Co. LLC. (St. Louis, MO, USA). Recombinant human soluble RANK ligand (sRANKL) was purchased from Peprotech Co. Ltd. (Rocky Hill, NJ, USA).

### 2.2. Bone-Resorbing Activity in Organ Cultures of Mouse Calvariae

Mouse calvariae were collected from newborn mice and precultured for 24 h in BGJb medium supplemented with 1 mg/mL bovine serum albumin (BSA) at 37 °C under 5% CO_2_ in the air. Calvariae were treated with LPS (1 µg/mL) and β-cry after preculture and further cultured for 5 days. The concentration of Ca in the cultured medium was measured using o-cresolphthalein complexone (OCPC).

### 2.3. Cultures of Primary Mouse Osteoblastic Cells

Primary osteoblastic cells (POBs) were isolated from newborn mouse calvariae after digestion with 0.1% collagenase (Roche Diagnostics GmbH, Mannheim, Germany) and 0.2% dispase (Roche Applied Science, Mannheim, Germany). POBs were cultured in α-modified MEM (αMEM) supplemented with 10% fetal bovine serum (FBS) at 37 °C under 5% CO_2_ in the air, as reported previously [5].

### 2.4. Measurement of the PGE_2_ Content in the Cultured Medium

The concentration of PGE_2_ in conditioned medium in POB cultures was measured using an enzyme immunoassay system (EIA) (GE Healthcare UK Ltd., Little Chalfont, UK). The cross-reactivity of the antibody in the EIA was calculated as follows: PGE_2_: 100%, PGE_1_: 7.0%, 6-keto-PGF_1α_: 5.4%, PGF_2α_: 4.3%, and PGD_2_: 1.0%.

### 2.5. Reverse Transcription-Quantitative PCR

Mouse POBs were cultured for 24 h in αMEM with 1% FBS with LPS (1 ng/mL) and β-cry (30 µM). Total RNA was isolated using ISOGEN (Nippon Gene Co., Ltd., Tokyo, Japan), and cDNA was prepared from RNA via reverse transcription. For real-time PCR, 5 µg of RNA was mixed with SsoAdvanced SYBR green supermix (Bio-Rad Laboratories, Inc., Hercules, CA, USA) and PCR primer pair, and real-time PCR was performed. The primer sequences for real-time PCR were as follows: mouse Rankl (NM_011613.3): 5′-aggctgggccaagatctcta-3′ (forward) and 5′-gtctgtaggtacg cttcccg-3′ (reverse), mouse Cox2 (NM_011198.4): 5′-gggagtctggaacattgtgaa-3′ (forward) and 5′-gtgcacatt gtaagtaggtggact-3′ (reverse), mouse mPges1 (NM_022415.3): 5′-gcacactgctggtcatcaag-3′ (forward) and 5′-acgtttcagcgcatcctc-3′ (reverse), mouse Ctsk (NM_007802.4): 5′-gcctagcgaacagattctcaa-3′ (forward) and 5′-cactgggtgtccagcattt-3′ (reverse), mouse β-actin (NM_007393.5): 5′-ccccattgaacatggcattg-3′ (forward) and 5′-acgaccagaggcatacagg-3′ (reverse). The results are shown as the relative fold expression normalized by β-actin compared with the control.

### 2.6. Dual-Luciferase Reporter Assay

Plasmid pNFκB-TA-Luc (0.4 µg) contained 4 tandem copies of the NF-κB consensus sequence with the firefly luciferase reporter gene (Clontech Laboratories, Inc., Mountain View, CA, USA), and the pGL4.74[hLuc/TK] plasmid (40 ng) contained the *Renilla* luciferase reporter gene (Promega Corp., Madison, WI, USA) as an internal control reporter vector. Both plasmids were transfected into mouse POBs using Lipofectamine 2000 (Thermo Fisher Scientific Inc., Waltham, MA, USA). The luciferase activity was measured using the Dual-Luciferase Reporter Assay System (Promega Corp.) with an ARVO MX multilabel/luminescence counter (Perkin Elmer Corp., Waltham, MA, USA).

### 2.7. Inhibitor of NF-κB Kinase (IKK) Activity Assay

The kinase activity of IKKβ was elucidated with or without β-cry (0.05–5 mM) using the Cyclex IKKα and β Assay/Inhibitor Screening Kit (CycLex Co. Ltd., Nagano, Japan) with IKKβ, IκBα, and anti-phospho-IκBα antibody.

### 2.8. Protein Structure Preparation

The three-dimensional X-ray crystal structure of IKKβ was obtained from a protein databank (PDB ID:4KIK, 2.83-Å resolution) [23]. For docking simulations, default parameters (H-atoms) were added into the protein structures using AutoDock Tools (Molecular Graphics Laboratory, La Jolla, CA, USA).

### 2.9. Ligand Structure Preparation

The chemical structure of β-cry was optimized using the online compound editor InDraw (http://in.indraw.integle.com; Integle Chemistry, Inc., Shanghai, China). All two-dimensional structures were converted into three-dimensional structures in the pdb format and saved in the mol format using Open Babel (http://www.openbabel.org/) [24].

### 2.10. Molecular Docking Studies

The protein–ligand molecular docking study was performed using AutoDock Vina [25]. Subsequently, AutoDock Vina was used to implement fast docking of the inhibitor ligand into the active pocket of both the IKKβ and kinase domains, which considered the flexibility and mobility of the ligand molecules and protein active-site residues, and used the Lamarckian genetic algorithm to fully explore the conformational space for the IKKβ inhibitor interactions. The rotational bonds of the protein were kept rigid, while those of the ligands were treated as flexible. The amino acids Leu21, Gly22, Tyr 23, Val29, Ala42, Lys44, Glu61, Val74, Met96, Glu97, Tyr98, Cys99, Gly102, Asp103, Glu149, Asn150, Val152, Ile165, Asp166, and the surrounding residues within a distance of 6.5 Å were defined as active sites.

### 2.11. Structural Visualization and Analyses

Pymol (http://apbs.sourceforge.net) was used for the analysis and visualization of protein–ligand interaction. Amino acids of active sites and the surrounding residues were set to be shown, while other parts were hidden.

### 2.12. RANKL-Induced Osteoclastogenesis in RAW264.7 Cells

RAW264.7 cells (2.5 × 10^4^ cells) were cultured in αMEM containing 10% FBS for 5 days with sRANKL (100 ng/mL) in the presence of β-cry (5–10 µM). The formed osteoclastic cells were stained for tartrate-resistant acid phosphatase (TRAP). TRAP-positive multinucleated cells containing three or more nuclei per cell were defined as osteoclasts.

### 2.13. Statistical Analyses

Data were analyzed using a one-way analysis of variance, followed by Tukey’s test for the post hoc analysis. All data are presented as the means ± standard deviation (SD), and all statistical analyses were performed using the IBM SPSS Statistics Ver.23 software program (Armonk, NY, USA).

## 3. Results

### 3.1. β-Cry Inhibited LPS-Induced Bone Resorption in Calvarial Organ Culture

**Figure 1:**
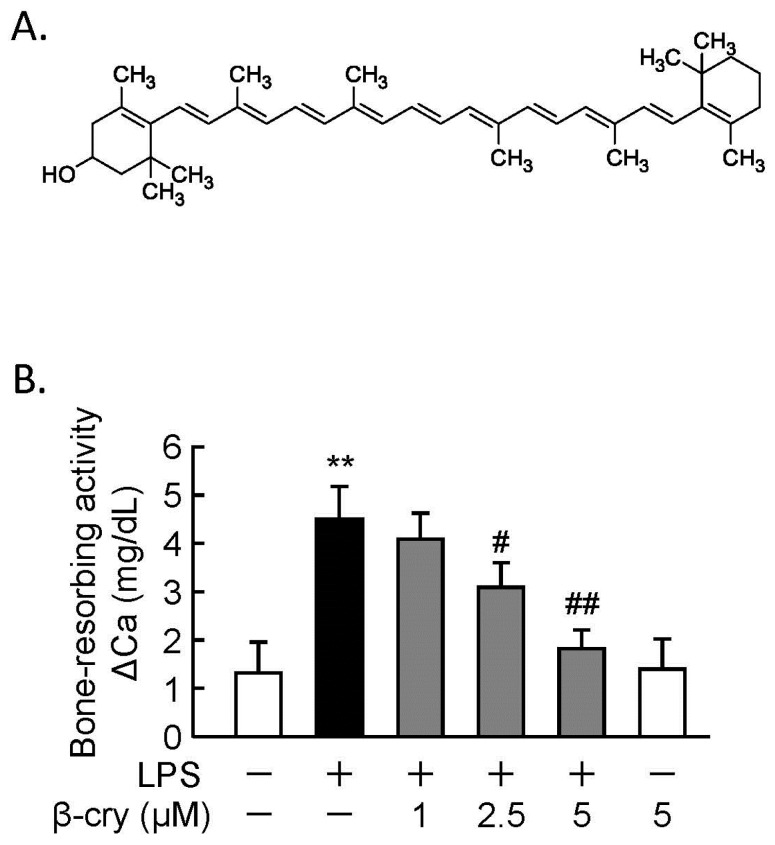
Beta-cryptoxanthin (β-cry) suppressed the lipopolysaccharide (LPS)-induced bone-resorbing activity. (**A**) The chemical structure of β-cry. (**B**) Mouse calvariae were cultured with LPS (1 µg/mL) and β-cry (1–5 µM) for five days. The Ca concentration in the conditioned medium was measured by the o-cresolphthalein complexone (OCPC) method, and the increase in Ca was evaluated as the bone-resorbing activity. Data are expressed as the mean ± SD for three independent cultures. Significant differences are indicated, ** *p* < 0.01 vs. control; ^#^ *p* < 0.05, ^##^ *p* < 0.01 vs. LPS.

The chemical structure of β-cry is shown in Figure 1A. We previously showed that β-cry suppressed LPS-induced osteoclast differentiation in cocultures of mouse bone marrow cells and POBs [22]. To examine the effects of β-cry on bone resorption, calvariae from newborn mice were cultured with or without LPS (1 µg/mL) and β-cry (5 µM). β-cry suppressed the LPS-induced bone resorbing activity in a dose-dependent manner in calvarial organ cultures, as seen in Figure 1B.

### 3.2. Effects of β-Cry on the PGE_2_ Production and NF-κB Signaling in Osteoblasts

**Figure 2:**
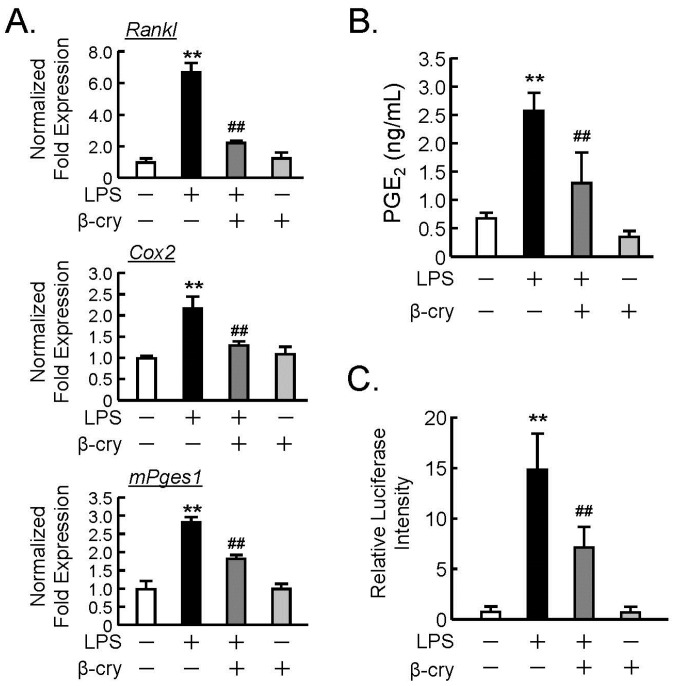
The effects of β-cry on the LPS-induced expression of cyclooxygenase (COX)-2, membrane-bound PGE synthase (mPGES)-1, and receptor activator of NF-κB ligand (RANKL) mRNA on the production of prostaglandin (PG)E_2_ in osteoblasts. Primary osteoblastic cells (POBs) were pre-treated with β-cry (5 µM) for 24 h and further cultured for 24 h in the presence of LPS (1 µg/mL) and β-cry (5 µM). (**A**) The mRNA expression of RANKL, COX-2, and mPGES1 was analyzed by RT-qPCR. Data are expressed as the mean ± SD of three replicated wells in triplicate. (**B**) The PGE_2_ concentration was measured in the cultured medium of POBs. (**C**) The transcription activity of NF-κB was measured with or without β-cry (5 µM). Plasmid pNFkB-TA-Luc (0.4 µg) and the pGL4.74[hLuc/TK] plasmid (40 ng) were transfected into POBs, and the luciferase activity was measured with the Dual-Luciferase Reporter Assay System. Data are expressed as the mean ± SD for 3–4 independent wells. Significant differences are indicated, ** *p* < 0.01 vs. control; ^##^ *p* < 0.01 vs. LPS.

We previously reported that β-cry suppressed the PGE_2_ production induced by LPS in the murine fibroblast cell line NIH3T3 and primary gingival fibroblasts [22]. In order to assess the effects of β-cry on osteoblasts, the mRNA expression of PGE-related genes was analyzed by real-time PCR. LPS augmented the mRNA expression of COX-2, mPGES1, and RANKL, whereas β-cry significantly suppressed the LPS-induced mRNA expression of these genes, as seen in Figure 2A. In addition, β-cry clearly inhibited PGE_2_ production in the culture of osteoblasts, as seen in Figure 2B. We further analyzed the NF-κB transcriptional activity using a reporter gene assay and found that β-cry significantly attenuated the LPS-induced NF-κB activity, as seen in Figure 2C.

### 3.3. β-Cry Inhibited IKKβ Activity Via ATP-Competitive Binding

**Figure 3:**
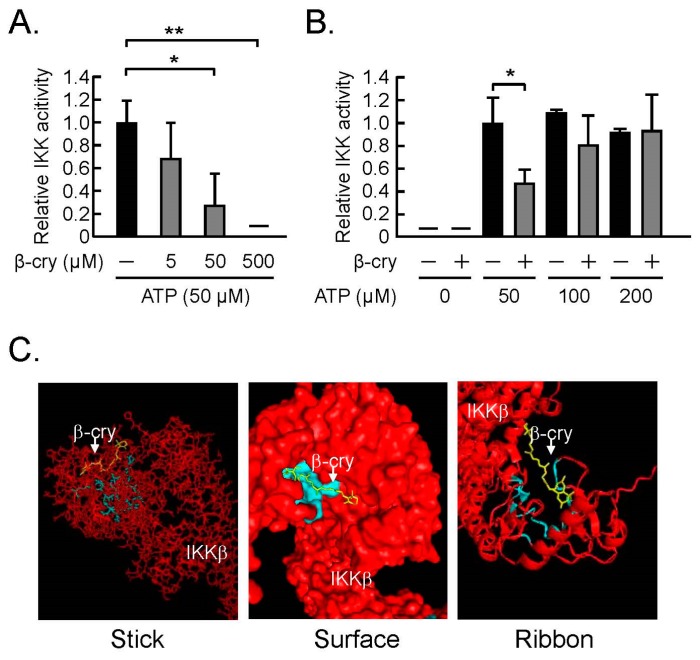
The direct action of β-cry on inhibitor of NF-κB kinase (IKK) activity in an in vitro assay. The IKK activity was elucidated by the IKK activity assay kit using IKKβ, IκBα, and anti-phospho-IκBα antibody. (**A**) The effects of β-cry on the IKK activity. (**B**) ATP was added to the IKK assay with β-cry, and the IKK activity was expressed as the percentage of the control without β-cry. Data are expressed as the mean ± SD for three independent wells. Significant differences are indicated, * *p* < 0.05, ** *p* < 0.01. (**C**) Predicted binding model of β-cry docked into a homology of IKKβ. The 3D images of the ligand binding model are shown as three models. The colors in the three images were indicated as follows: yellow (β-cry), red chain (IKKβ), and light blue (active site in IKKβ).

Kim et al. [26] suggested that flavonoid binds to the ATP-binding pocket of IKKβ to suppress its kinase activity and attenuate NF-κB activation. Our data similarly showed that β-cry inhibited the recombinant IKK activity in a dose-dependent manner, as seen in Figure 3A, and this effect of β-cry was also canceled by ATP in a dose-dependent manner, as seen in Figure 3B. In order to examine the possible binding model of β-cry to IKKβ, β-cry was subjected to molecular docking using a homology model of IKKβ. In Figure 3C, the 3D images of the ligand binding model are shown as three models. The colors in the three images were indicated as follows: yellow (β-cry), light blue (active site), and red (IKKβ). The amino acids Leu21, Gly22, Tyr 23, Val29, Ala42, Lys44, Glu61, Val74, Met96, Glu97, Tyr98, Cys99, Gly102, Asp103, Glu149, Asn150, Val152, Ile165, Asp166, and the surrounding residues within a distance of 6.5 Å were defined as active sites. β-cry was positioned in the catalytic center, a pocket-like structure of the assumed binding site (Figure 3C). These data suggest that β-cry binds to the ATP-binding pocket of IKKβ to suppress its kinase activity, leading to attenuation of the NFκB pathway.

### 3.4. β-Cry Directly Suppressed sRANKL-Induced Osteoclast Differentiation

**Figure 4:**
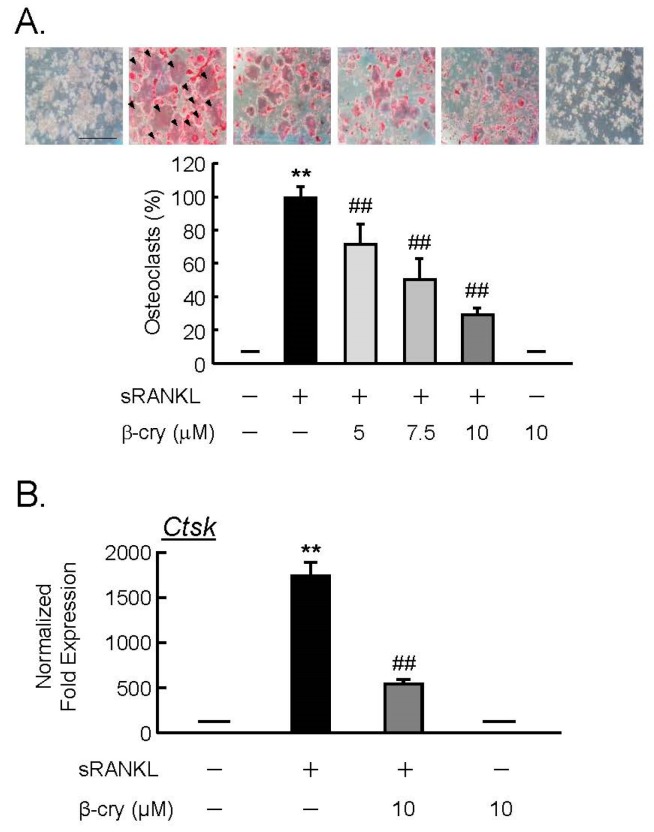
β-cry inhibits the soluble RANK ligand (sRANKL)-induced osteoclast differentiation and expression of cathepsin K (Ctsk) mRNA in RAW264.7 cells. RAW264.7 cells, which are mouse macrophages, were cultured with sRANKL (100 ng/mL) and β-cryptoxanthin (5–10 µM) for four days. (**A**) Osteoclasts were stained for tartrate-resistant acid phosphatase (TRAP), and the percentage of TRAP-positive multinucleated cells was measured. Data are expressed as the mean ± SD for eight independent wells. Arrowheads indicate TRAP-positive multinucleated osteoclasts. Bar = 500 µm. (**B**) Total RNA was extracted and reverse transcribed into cDNA. cDNA was amplified, and the mRNA expression of cathepsin K (Ctsk) was measured by RT-qPCR. Data are expressed as the mean ± SD of three replicated wells in triplicate. Significant differences are indicated, ** *p* < 0.01 vs. control; ^##^ *p* < 0.01 vs. sRANKL.

In order to test the direct action of β-cry on osteoclast precursor cells, RAW264.7 cells were cultured for four days with sRANKL (100 ng/mL) and β-cry (5–10 µM). β-cry suppressed the RANKL-induced osteoclast differentiation in a dose-dependent manner, as seen in Figure 4A. The mRNA expression of cathepsin K (*Ctsk*) was elevated by RANKL in RAW264.7 cells, but this increase was repressed by adding β-cry to the real-time PCR analysis, as seen in Figure 4B.

## 4. Discussion

β-cry is known to be abundantly found in Japanese mandarin oranges and has been reported to exert various beneficial effects, such as antioxidant and cancer-preventive effects [10,11,12]. Japanese mandarin oranges (*Citrus unshiu*) contain high levels of β-cry and are produced in Japan. Sugiura et al. [27] reported that a daily intake of one or more *Citrus unshiu* increased the concentration between 0.5 to 3.5 µM of serum β-cry in healthy Japanese individuals. They also reported that β-cry is easily accumulated in various tissues compared with β-carotene [28]. Several reports have shown that β-cry exhibited preventive effects on estrogen deficiency-induced bone loss, rheumatoid arthritis, and osteoarthritis in in vivo studies [14,29,30]. Our previous study showed that β-cry inhibited LPS-induced osteoclast differentiation in cocultures of mouse bone marrow cells and POBs and restored the bone loss of mandibular alveolar bone induced by local injection of LPS in a mouse model of periodontitis [22]. This study also showed that 5 µM of β-cry did not exhibit cell cytotoxicity in osteoblasts [22]. In the present study, we showed that β-cry inhibited the COX-2- and mPGES-1-mediated PGE_2_ production in osteoblasts and further suggested that IKKβ was a novel molecular target of β-cry in LPS-induced osteoclast differentiation. Therefore, it is possible that β-cry may regulate inflammatory response in various cells, but further studies are needed to clarify this point.

Osteoblasts perform dual roles in bone formation and bone resorption. The expression of RANKL is induced by inflammatory molecules, such as LPS, and is essential for inflammatory bone resorption. NF-κB is the key transcription factor associated with the regulation of COX-2 and mPGES1 after LPS stimulation [31,32]. We previously reported that the lack of PGE_2_ production in mPGES1-deficient mice (mPges1^−/−^) attenuated the LPS-induced inflammatory bone destruction of alveolar bone [5]. In the present study, we found that β-cry suppressed the mRNA expression of PGE_2_ synthases, COX-2, and mPGES-1, as well as PGE_2_ production via the attenuation of NF-κB activity in osteoblasts, leading to the downregulation of RANKL expression. Along with our previous finding that β-cry inhibited LPS-induced osteoclast differentiation in cocultures of mouse bone marrow cells and POBs [22], the present results suggest that β-cry inhibits osteoclast differentiation and bone resorption by downregulating the PGE_2_-mediated RANKL expression in osteoblasts and by direct action on osteoclast precursors to suppress osteoclast differentiation through inhibiting NF-κB activity. However, it was also reported that β-cry stimulated cell growth, differentiation, and mineralization via the upregulation of bone formation genes, including Runx2, in the mouse osteoblastic cell line MC3T3-E1 [15,16,17]. Therefore, β-cry may act on both bone resorption and bone formation in bone tissues.

Uchiyama et al. [20,21] reported that β-cry suppressed the formation of osteoclast-like cells induced by bone-resorbing factors, such as parathyroid hormone (PTH), PGE_2_, 1,25-dihydroxyvitamin D_3_, LPS, and TNFα, and induced apoptotic cell death through the upregulation of apoptosis protease-associating factor (Apaf)-2 and caspase-3 in mouse marrow cultures and osteoclastic cell cultures. Ozaki et al. [14] reported that β-cry inhibited sRANKL-induced osteoclastogenesis, downregulated the mRNA expression of TRAP, and attenuated the activity of NF-κB but not AP-1 and NFATc1 in Raw264.7 cells. In the present study, we found that β-cry suppressed sRANKL-induced osteoclast differentiation and downregulated the mRNA expression of cathepsin K in cultures of RAW264.7 cells. Therefore, β-cry may act directly on osteoclast precursor cells to inhibit their differentiation into mature osteoclasts.

Since β-cry is a provitamin A, the effects of β-cry might be attributed to that of vitamin A (retinol). However, a previous study showed that retinol was not detected in RAW264.7 cells treated with β-cry [11]. Uchiyama et al. [15] suggested that MAPK or protein kinase C (PKC) are potential targets of β-cry in osteoblasts and speculated that β-cry could bind to orphan receptors to exert its biological functions. We previously suggested that lutein, a carotenoid, could directly inhibit the kinase activity of IKK [33]. IKK complex, an enzyme complex consisting of the catalytic kinases IKKα and IKKβ as well as a regulatory subunit NEMO, is a signal component of NF-κB signaling. IKK complex stimulates NF-κB translocation by the phosphorylation and degradation of inhibitor of NF-κB (IκB). The IKK complex is activated by various inflammatory responses, including proinflammatory cytokines, bacterial LPS, RANKL–RANK signaling, viral infection, and stress-induced responses [34,35,36]. We examined the effects of β-cry on IKKβ activity with an in vitro assay and found that β-cry directly suppressed the IKKβ activity. Kim et al. [26] suggested that luteolin, a flavonoid, could bind to the ATP-binding pocket of IKKβ to inhibit its activity and attenuate NF-κB activation. Our present study indicated that excess ATP could competitively inhibit the effects of β-cry on IKKβ activity. These findings suggest that β-cry inhibits IKKβ activity via competition with ATP-binding pocket, leading to the attenuation of NFκB activation and osteoclast differentiation. Sahin et al. [12] have shown that β-cry suppressed nuclear localization of NFκB in various cells in liver and adipose tissues. Further studies are needed to define the effects of β-cry on the nuclear localization of NFκB in both osteoblasts and osteoclasts. 

In the present study, we have suggested that IKKβ might be the novel target of β-cry in osteoclast differentiation. Since the IKK complex is involved in both LPS–TLR4–IKKs–NFκB signaling and RANKL–RANK–IKKs–NFκB signaling, β-cry may inhibit the LPS-induced RANKL expression in osteoblasts and RANKL-induced osteoclast differentiation in osteoclast precursor cells. These data are helpful for understanding the molecular mechanism of action of β-cry, which may be a potential natural compound for the prevention of inflammatory bone loss.
